# Palatal Swelling: Exploring an Elusive Diagnostic Dilemma

**DOI:** 10.7759/cureus.45250

**Published:** 2023-09-14

**Authors:** Preksha S Mishra, Namish Batra, Dharmesh Vasavada

**Affiliations:** 1 Oral and Maxillofacial Surgery, Manubhai Patel Dental College, Vadodara, IND; 2 Oral Pathology and Microbiology, Manubhai Patel Dental College, Vadodara, IND

**Keywords:** palatal swelling, salivary gland neoplasm, mucoepidermoid carcinoma (mec), low grade malignancy, hard palate

## Abstract

Mucoepidermoid carcinoma is a locally invasive tumor of the major and minor salivary glands. A 29-year-old male patient reported a complaint of slow-growing, painless, non-ulcerated palatal swelling. On clinical evaluation, the swelling appeared benign; hence, the complete excision of the lesion was carried out under general anesthesia, with closure by reconstruction with a partial-thickness flap. Healing was uneventful. The histopathological evaluation revealed low-grade mucoepidermoid carcinoma. This case report aims to highlight the importance of proper clinical and histopathological evaluation to rule out malignancy, as mucoepidermoid carcinoma can have variable presentations and mimic various benign salivary gland lesions, similar to the occurrence in the present case.

## Introduction

Mucoepidermoid carcinoma, a malignant epithelial tumor of the salivary gland, represents 29%-34% of malignant tumors observed in major and minor salivary glands. It accounts for 5% of all salivary gland tumors. It shows slight female predilection, occurring usually in the third or fifth decades of life [1]. Mucoepidermoid carcinoma consists of mucous, epidermoid, and intermediate cells. The intermediate cells are considered to be progenitors for mucous and epidermoid cells. Previously, it was considered to be a benign tumor. WHO classified it as a malignant neoplasm in 1990 and renamed it mucoepidermoid carcinoma [2]. It is most commonly associated with the parotid gland and is considered the most common site of its occurrence. Other intraoral sites where it is commonly found are the hard palate, tongue, buccal mucosa, and retromolar area [1]. The aim is to highlight the importance of proper preoperative diagnosis since mucoepidermoid carcinoma mimics most minor salivary gland tumors, typically appearing as asymptomatic swellings that are fluctuant and have a blue or red color and can be easily mistaken clinically for other minor salivary gland lesions. 

## Case presentation

**Figure 1:**
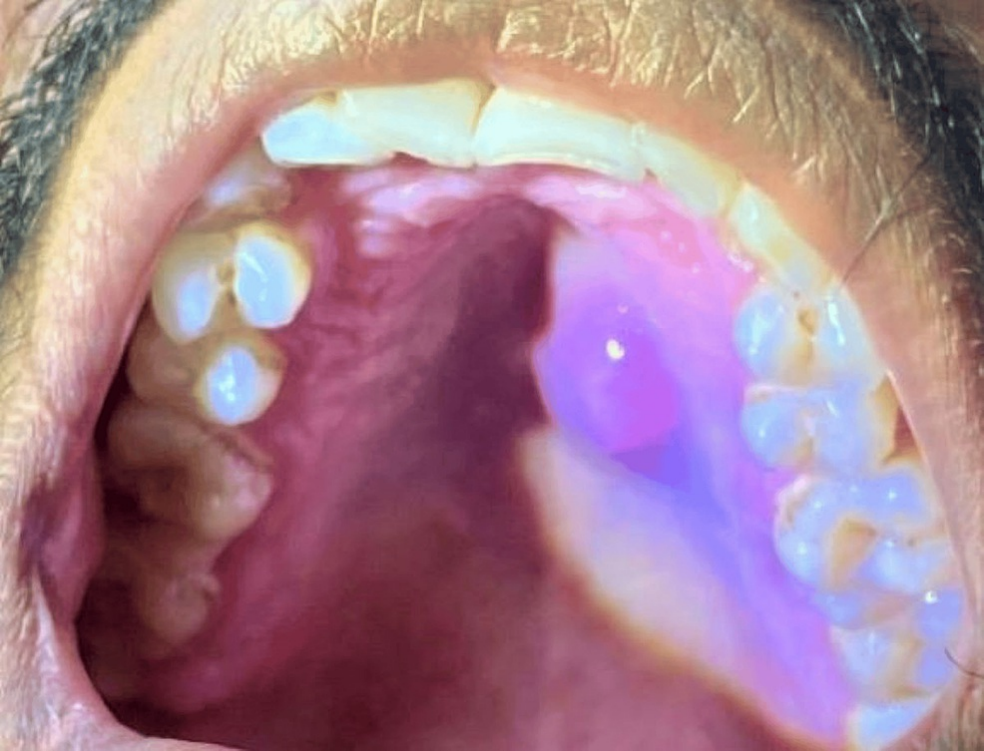
Preoperative image showing the palatal lesion

A 29-year-old male patient reported having an intraoral swelling on the left side of the hard palate for two years, which gradually increased in size, and complained of discomfort. The patient did not give any history of pain or fever and had no complaint of bleeding or pus discharge. The patient had no relevant medical, dental, family, or habit history. On inspection, an ovoid, solitary, well-defined swelling with regular borders extended from the region distal to 22 up to the region mesial of 26. Anteroposteriorly, it extended 3-4 cm away from the incisive foramen and 6-7 cm away from the soft palate. It was located 1 cm away from the mid-palatine raphe and 3-4 cm away from the buccal aspect of 26. The swelling was approximately 3-4 cm in diameter. The overlying mucosa was intact, with no signs of ulceration (Figure 1). On palpation, the swelling was non-tender, soft, and non-fluctuant and showed no signs of lymphadenopathy.

Investigation

Based on the patient's symptoms and history, the lesion was initially suspected to be a pleomorphic adenoma. This was due to the patient's history of gradually enlarging painless swelling and mild discomfort while masticating, which aligns with typical pleomorphic adenoma characteristics. Considering the bluish-red appearance of the lesion, another provisional diagnosis that could be given was capillary hemangioma.

The differential diagnosis that could be given was mucoepidermoid carcinoma and other vascular lesions.

To confirm the diagnosis, radiographic imaging was carried out (CT scan and sectional cone beam computed tomography [CBCT]), which did not give any evidence of a cystic lesion; thus, the need for pre-operative cytology was ruled out.

**Figure 2:**
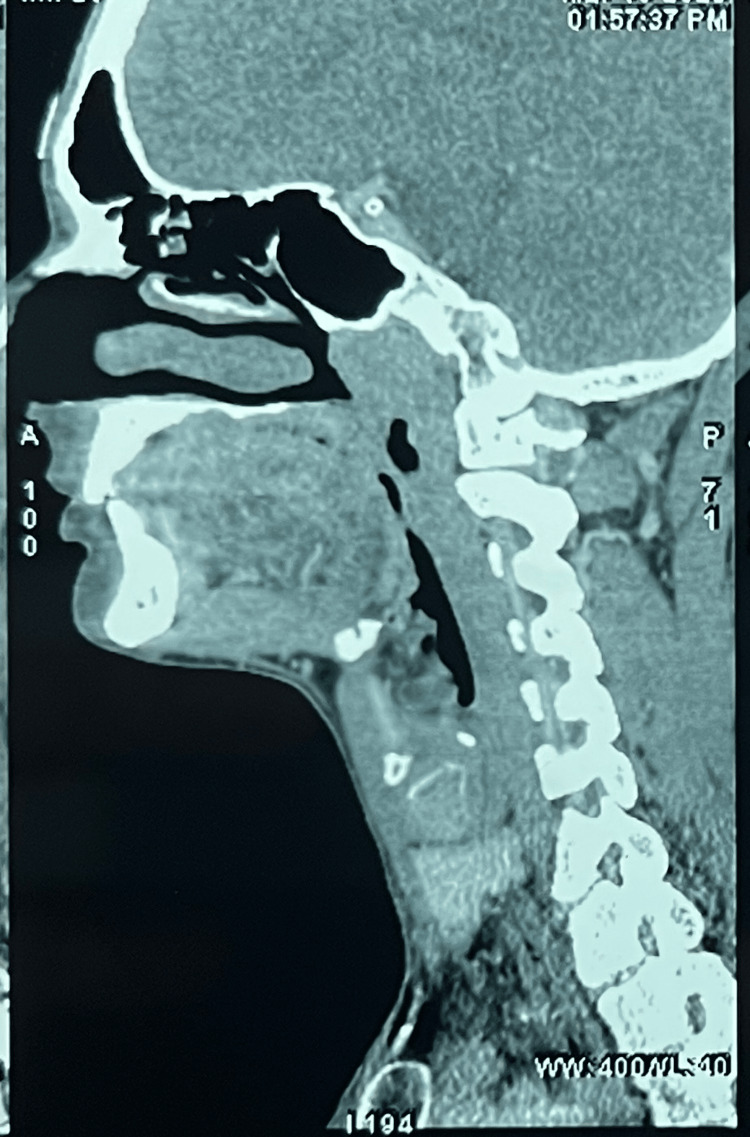
CT scan image showing the scalloping of the palatal cortex

**Figure 3:**
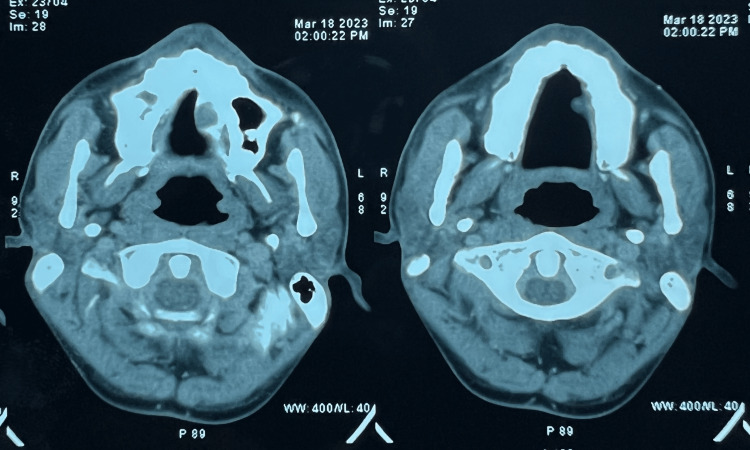
CT scan image showing the soft tissue lesion

A head and neck CT scan was carried out, which revealed an ill-defined soft tissue density lesion measuring 1.7x 1.6x 1.2cm (AP X T X CC) involving the hard palate on the left side. Scalloping with significant thinning of bone was evident. No intranasal or contralateral extension was seen (Figures 2, 3).

**Figure 4:**
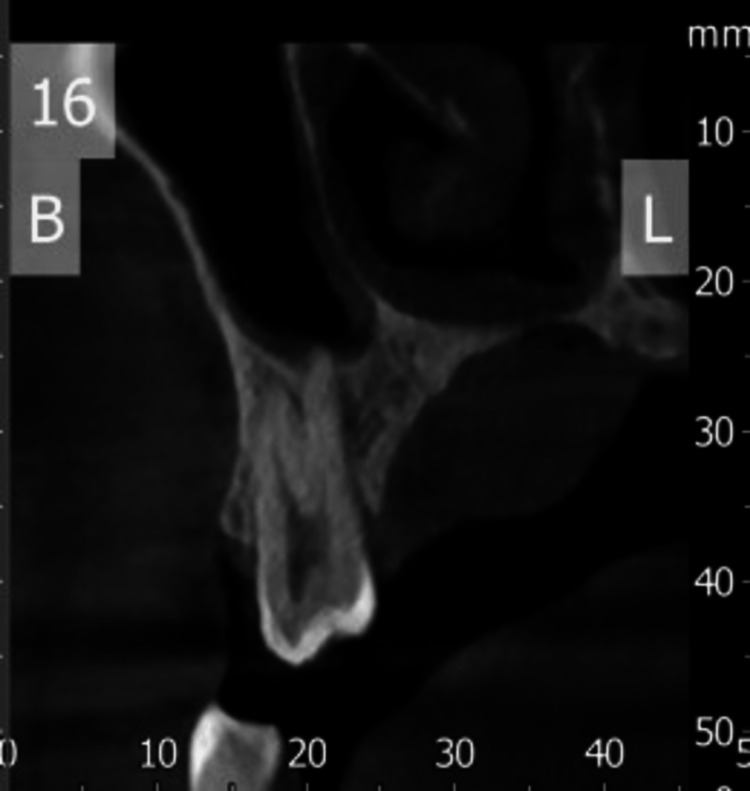
Sectional CBCT section showing scalloping of the palatal cortex

The rest of the oral cavity and tongue appeared normal. No involvement of the bilateral parotid, submandibular, or thyroid glands was seen. To further confirm the diagnosis, a sectional CBCT was done (Figure 4), which revealed a soft tissue shadow covering almost half of the palate. Thinning of the palatal cortex was seen. In the radiographic examination, a hypodense shadow is seen, which is suggestive of a salivary gland lesion but not a cystic lesion.

Treatment

**Figure 5:**
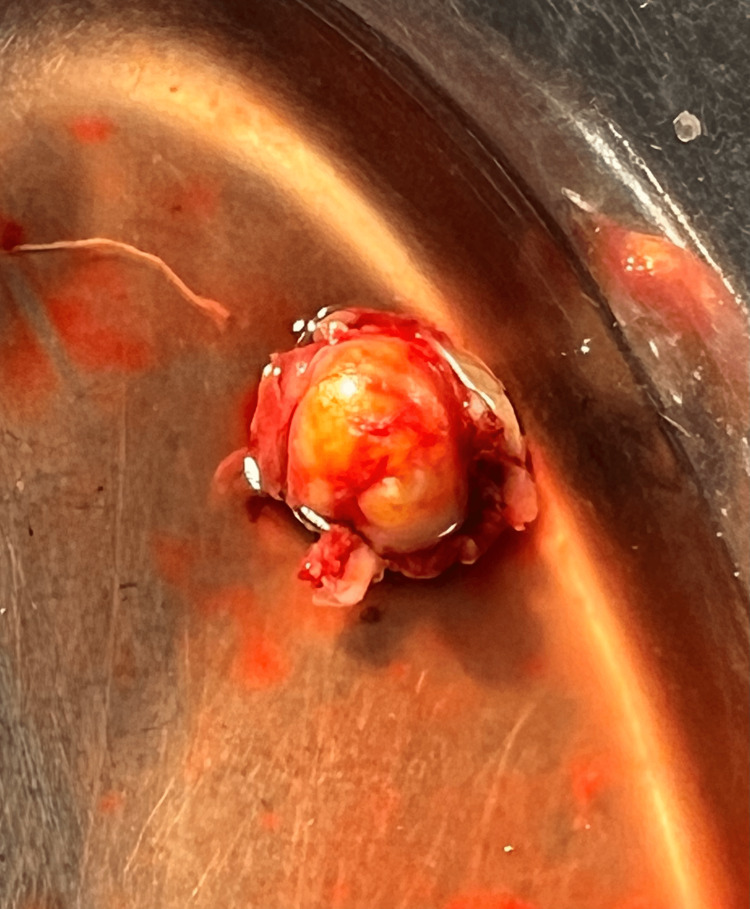
Photo showing the excised palatal lesion

A pre-surgical evaluation was carried out, and a complete excision of the tumor from the left mid-palatal region was done under general anesthesia. A palatal crevicular incision was made, and the corresponding palatal flap was raised. A complete excision of the lesion was carried out (Figure 5). A feeder vessel from the greater palatine artery was ligated during the excision. Palatal reconstruction was done by using a partial-thickness flap on the contralateral side. The surgical site was closed with 4-0 vicryl. This surgical approach was carried out to ensure there was no involvement of other minor salivary glands. The complications that could be expected were postoperative hemorrhage and infection of the surgical site, but fortunately, none were reported. Considering the high recurrence rate of mucoepidermoid carcinoma, the recurrence of the lesion cannot be prevented despite the complete excision of the lesion. It can also be considered a surgical complication; therefore, the patient has been kept on follow-up. The pathology has a fair prognosis. 

**Figure 6:**
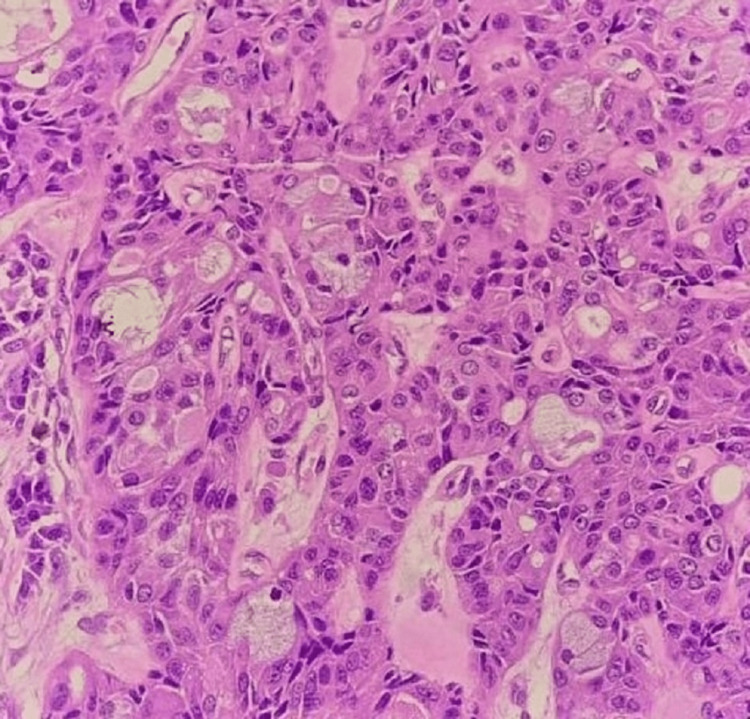
A high-power view (40x) shows intermediate cells and mucous cells within the stroma. A few myoepithelial cells are also seen.

**Figure 7:**
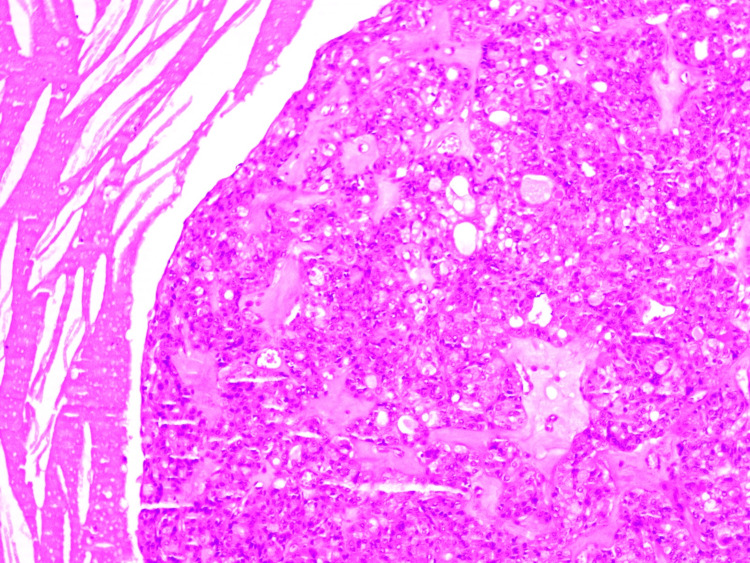
A high-power view (10x) shows squamous, mucous, and intermediate cells arranged within the stroma. Predominantly mucous cells are seen, with few clear cells.

Histopathological examination revealed many cystic spaces containing cholesterol-cleft nests of tumor cells composed of glandular mucinous cells, intermediate cells, and squamous epithelium. Evidence of infiltrative tumor nests in the adjacent stoma was seen. No evidence of perineural invasion, high-grade nuclear anaplasia, or prominent mitotic activity confirmed the diagnosis of low- to intermediate-grade mucoepidermoid carcinoma (Figures 6, 7).

## Discussion

Salivary gland tumors are relatively uncommon compared to other tumors and account for less than 2% of all human tumors [3]. Although neoplasms of the salivary gland are uncommon, they particularly interest histopathologists as they show varied histopathological characteristics [2]. Mucoepidermoid carcinoma has an incidence of approximately 3/1,000,000 people, with slight predilection in females. Salivary gland carcinoma comprises only 3%-5% of all head and neck malignancies. Overall, mucoepidermoid carcinoma accounts for 2.8%-15.5% of all salivary gland tumors, 1%-10% of all major salivary gland tumors, and 6.5%-41% of minor salivary gland tumors. Mucoepidermoid carcinoma can be seen in any age group but most commonly in middle age (35-65 years), with an average age of 47 years [4]. The etiology of mucoepidermoid carcinoma is still not very clear, but it is considered to occur due to multiple genetic mutations. It is specifically associated with t(11;19)(q21;p13) translocation. This translocation creates the MECT1-MAML2 fusion protein, which disrupts the Notch signaling pathway [5]. Mucoepidermoid carcinoma appears as asymptomatic swelling and shows a variety of biological behaviors and a variable natural history [6]. A high-grade mucoepidermoid carcinoma may, at times, give the appearance of squamous cell carcinoma if it is present clinically with an ulcerated surface along with the destruction of the underlying bone. It might also spread to the nasal cavity and maxillary antrum, resembling nasal and sinus carcinomas [2]. Clinically, the symptoms of a low-grade mucoepidermoid carcinoma are usually seen as a swelling that is slow-growing, painless, and fixed in nature, which is widely variable in the duration of occurrence. Immediately before clinical presentation, it may go through a phase of accelerated growth. Patients mainly complain of dysphagia [7]. Low-grade mucoepidermoid carcinomas are, at times, clinically manifested as bluish-red and fluctuant lesions resembling mucocele and other vascular lesions, leading to difficulty in proper clinical diagnosis, as seen in the present case. They occasionally invade the underlying bone, but this is not uncommon. Thinning of the palatal cortex is seen in the current case. Other times, in cases of low-grade malignancy, mucoepidermoid carcinoma usually appears as a slowly enlarging, painless mass that resembles pleomorphic adenoma. However, unlike the pleomorphic adenoma, the low-grade mucoepidermoid carcinoma seldom exceeds 5 cm in diameter, is not completely encapsulated, and often contains cysts that may be filled with a viscid mucoid material [1]. The other most common entity is the palatal space abscess, mostly found in children, which is characterized by a tender, diffuse, and erythematous swelling of sudden onset. Adenoid cystic carcinoma and adenosquamous carcinoma can be differentiated by histological examination. Various differential diagnoses based on the clinical findings are discussed in the Table 1. Table 2 shows the radiographical findings for various salivary gland lesions.

**Table 1:** Differential diagnosis based on clinical findings.

Clinical Findings	Rule In	Rule Out
Slow growing, enlarging, painless, palatal swelling	Monomorphic Adenoma	Adenoid Cystic Carcinoma
	Pleomorphic Adenoma	
	Mucoepidermoid Carcinoma (Low grade)	Fibrosarcoma
	Polymorphous Low Grade Adenoid Cystic Carcinoma (PLGA)	
Non- Ulcerated Lesion	Monomorphic Adenoma	Adenoid Cystic Carcinoma
	Pleomorphic Adenoma	
	Mucoepidermoid Carcinoma (Low Grade)	Fibrosarcoma
	Polymorphous Low Grade Adenoid Cystic Carcinoma (PLGA)	

**Table 2:** Radiographical findings for various salivary gland lesions.

Salivary Gland Neoplasm	Radiological Findings
Pleomorphic Adenoma	Smoothly marginated or lobulated homogeneous soft tissue density globular mass.
	A few foci of calcification are common.
Mucoepidermoid Carcinoma	Low-grade tumors appear as well-circumscribed masses, usually with cystic components. The solid components are enhanced, and calcification is sometimes seen. They have appearances similar to pleomorphic adenoma [8].
	High-grade tumors, on the other hand, have poorly defined margins, infiltrate locally and appear solid [8].
Adenoid Cystic Carcinoma	Low-grade tumors tend to be well-defined [9].
	High-grade tumors appear infiltrative [9].
	Adenoid cystic carcinomas are frequently associated with perineural spread (via cranial nerve VII), which is well appreciated on MRI [9].

As mentioned earlier, the CT scan did not show any evidence of perineural invasion; hence, adenoid cystic carcinoma was ruled out. Furthermore, no evidence of a cystic or infiltrative lesion was evident, which pointed more towards pleomorphic adenoma.

Hence, based on radiographic evidence, a working diagnosis of pleomorphic adenoma was established.

From the present case, the learning point that can be established is that accurate diagnosis is difficult solely based on clinical features, as most benign and malignant salivary gland tumors resemble each other grossly if seen early in their clinical course. In view of this fact, these tumors are treated based on histological and local findings, making a correct histopathological diagnosis obligatory [3]. Adequate histological material should be harvested to perform a complete evaluation of the morphology and cytology of the tumor to ensure the accuracy of the diagnosis.

The WHO defines mucoepidermoid carcinoma as "a malignant glandular epithelial neoplasm characterized by mucous, intermediate, and epidermoid cells with columnar, clear cells and oncocytoid features" [10]. Mucoepidermoid carcinoma can show a diverse histopathological morphology based on the predominant cell type and pattern. The clinical behavior of this tumor is usually predicted by its histological grade. Intermediate cells, a group of highly prolific basaloid cells, are more important in recognizing mucoepidermoid carcinoma. It is graded into low, intermediate, and high grades. A low-grade MEC shows a predominance of mucous-secreting cells. An intermediate-grade tumor comprises solid as well as cystic and epidermoid cells. The high-grade tumor predominantly consists of epidermoid cells with very few mucinous cells [2]. Treatment of mucoepidermoid carcinoma depends on its aggressiveness and the extent of its spread. When the tumor is confined to the palatal mucosa with intact periosteum, wide excision of the lesion along with the underlying mucoperiosteum is advised [11]. If the lesion is extensive, leading to the erosion of the underlying bone, it is treated with a complete excision of the lesion and the underlying bone. In cases where early detection is not possible, the lesion may show extensive spread involving the maxillary sinus and nasal cavity, which would further demand extensive surgery comprising palatectomy, maxillectomy, and reconstructive surgery. Conservative surgical excision is the primary treatment for mucoepidermoid carcinoma of minor salivary glands, along with long-term follow-up with the patient.

## Conclusions

Early detection, proper diagnosis, and prompt treatment result in a better prognosis for the patient. Giving a final diagnosis solely based on clinical findings is difficult, as mucoepidermoid carcinoma has variable representations. A complete histopathological evaluation by harvesting adequate histopathological material should be carried out to ensure a proper diagnosis. The treatment usually comprises a complete excision of the lesion, depending on its aggressiveness and the extent of its spread. In this case, the complete excision of the lesion was carried out under general anesthesia, with closure by reconstruction with a partial-thickness flap. Owing to its high recurrence rate, keeping the patient on a long-term follow-up is necessary.
